# Laboratory diagnostics, phylogenetic analysis and clinical outcome of a subcutaneous *Mycoleptodiscus indicus* infection in an immunocompetent cat

**DOI:** 10.1186/s12917-019-2132-1

**Published:** 2019-10-21

**Authors:** Grazieli Maboni, Paula Krimer, Rodrigo Baptista, Ana Lorton, Christina Anderson, Susan Sanchez

**Affiliations:** 1 Athens Veterinary Diagnostic Laboratory, University of Georgia, Athens, GA USA; 2 Center for Tropical and Emerging Global Diseases, University of Georgia, Athens, GA USA; 3 Institute of Bioinformatics, University of Georgia, Athens, GA USA; 4 Animal Hospital of East Cobb, Marietta, GA USA; 5 Department of Infectious Diseases, College of Veterinary Medicine, University of Georgia, Athens, GA USA

**Keywords:** *Mycoleptodiscus indicus*, Feline, Subcutaneous infection, Cytology, Fungal culture, Phylogenetic analysis

## Abstract

**Background:**

*Mycoleptodiscus indicus* is a dematiaceous hyphomycete fungus found on plant leaves. It has been rarely reported as a cause of human or animal disease, possibly because it is difficult to culture and identify from clinical specimens. Infections are presumably acquired by traumatic implantation.

**Case presentation:**

An 8-year-old non-immunosuppressed cat from Georgia, USA, presented with a left front leg swelling without lameness. Cytology from a fine needle aspirate revealed pyogranulomatous inflammation with both cytoplasmic and extracellular fungal elements. There were septate hyphae with irregularly sized segments, non-staining uneven walls, and rounded yeast-like forms from which longer hyphae arose in a hub-and-spoke pattern. A mold was isolated on agar from a fine needle aspirate collected 1 week later and identified as *M. indicus* by morphology, DNA sequencing and phylogenetic analysis. The cat recovered completely and uneventfully with antifungal treatment.

**Conclusions:**

We report a previously undescribed presentation of *M. indicus* causing a subcutaneous infection in a cat with successful antifungal treatment. In this study we highlight the potential of *M. indicus* to infect immunocompetent animals, and the veterinary medical community should be aware of its unusual but characteristic clinical, microbiological and cytologic presentation.

## Background

*Mycoleptodiscus spp.* are dematiaceous hyphomycete fungi that include plant pathogens, opportunistic animal infections, saprobic and endophytic fungi [1–3]. Currently, the *Mycoleptodiscus* genus comprises 18 species [2]. *M. indicus* is generally found on leaves of a number of different host plants in tropical and subtropical environments [1, 3]. In certain plant species, such as *Zamia* spp. (an American cycad), *M. indicus* has been isolated from lesions on leaves [1]. Other *Mycoleptodiscus* species have been reported to cause disease in economically important plants [1, 4]. Although *M. indicus* has been widely recognized as a plant pathogen, it has rarely been reported to be associated with human or animal disease. The limited number of clinical reports might be attributed to the fastidious nature of *M. indicus*, which can be difficult to identify in clinical specimen cultures. In addition, antifungal treatment for these cases is reported to be challenging [5]. *M. indicus* is typically found in immunocompromised human hosts causing subcutaneous infections [5, 6], cellulitis and sometimes myositis [6]. In an immunocompetent healthy man, *M. indicus* was reported to cause septic arthritis [7]. Infections are presumably acquired by traumatic implantation [8], with plants implied as potential sources of infection [7]. In veterinary medicine, *M. indicus* has been reported in one case of an immunosuppressed dog, presenting with dermal excoriations and draining tract from a swollen leg [8]. To our knowledge, this is the first report in a cat to provide detailed identification of this microorganism via cytology, fungal culture, DNA sequencing, phylogenetic analysis, and to report treatment with a successful clinical outcome.

## Case presentation

An indoor-outdoor 8-year-old, 6.9 kg male neutered domestic long-haired cat presented with a left front leg swelling. The animal was receiving topical and oral flea preventative monthly with Spinetoram (Feline Cheristin, Elanco, IN, USA) and was current on vaccinations for feline herpesvirus, calicivirus, panleukopenia virus, leukemia virus, and rabies. The cat had a history of infrequent seizures, less than one per year. Over the 18 months prior to presentation, he had been prescribed antibiotics and short courses of steroids twice for two different unrelated problems. In January 2017, he was administered cefpodoxime proxetil 50 mg daily for 14 days for chin pyoderma, and a month later treated for eosinophilic lip ulcers with a single 20 mg methylprednisolone acetate injection. Both lesions resolved within 2 months. Approximately in October 2017, he presented with a swollen, necrotic and purple digital lesion on the right hind paw, and slight weight loss (0.2 kg). This lesion healed after a single subcutaneous injection of 4 mg dexamethasone and 2 weeks of oral amoxicillin/clavulanic acid 25 mg administered twice daily and palliative wound management.

When the patient presented in August 2018, his front leg swelling began distal to the elbow and terminated approximately 1/3 from the distal radius. He had not traveled and there was no known trauma to the area. Rectal temperature was 37.7 °C. The cat did not have any other abnormal clinical signs, was eating well and was not lame. A fine needle aspiration from the left front leg recovered fluid and the small amount of material aspirated was spread onto five glass slides. The unstained direct smears were submitted to the Athens Veterinary Diagnostic Laboratory (University of Georgia, USA) for cytologic evaluation. A single injection of cefovecin 52 mg (Convenia, Zoetis, NJ, USA) was given and the cat was sent home for monitoring.

**Fig. 1:**
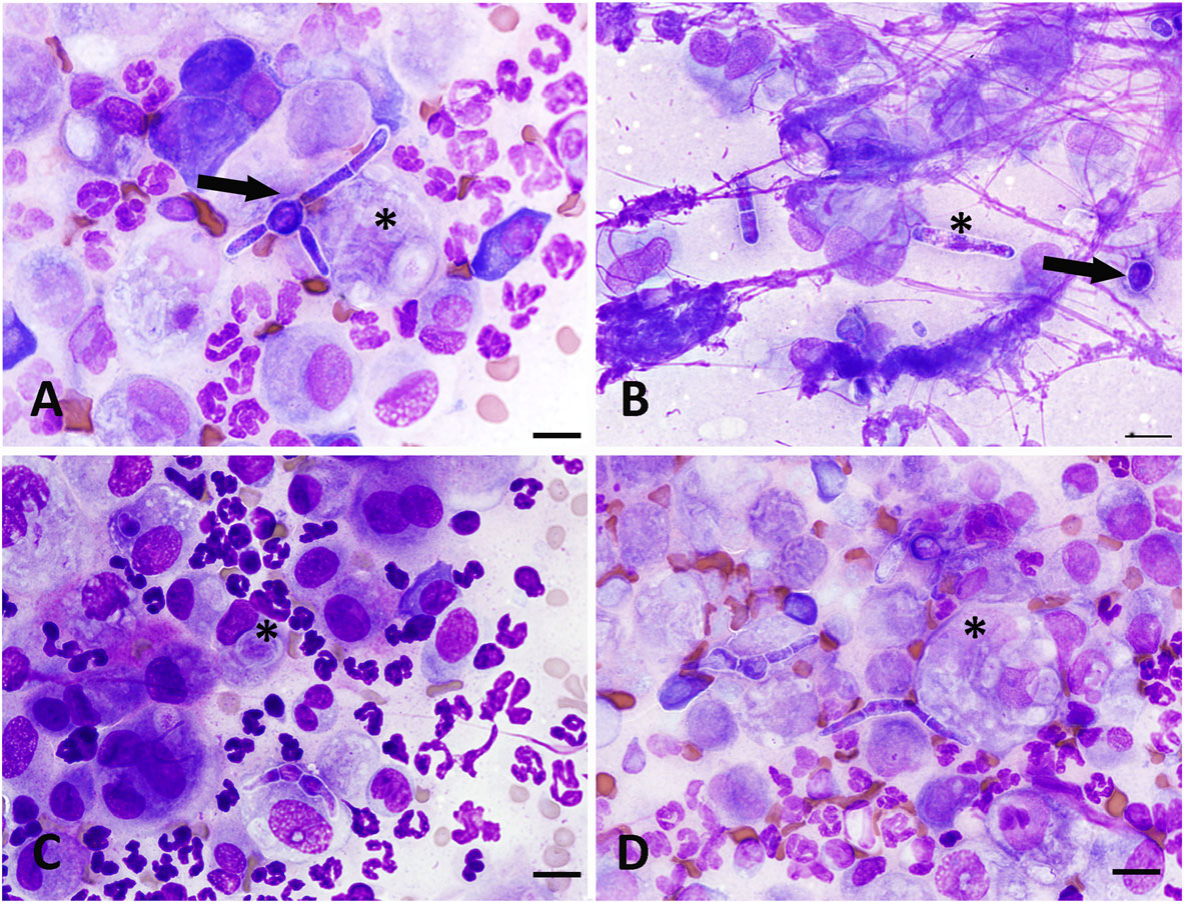
Cytological features of *Mycoleptodiscus indicus* subcutaneous infection in an 8-year-old cat, modified Wright-Giemsa stain. **a** Pyogranulomatous inflammation with both intralesional and extracellular fungal elements (asterisk). There are septated hyphae with irregularly sized segments, thin non-staining uneven walls, and some rounded yeast-like forms. Note the central yeast with radiating spokes of hyphae at the arrow (hub and spoke pattern). **b** Segments vary markedly in size, including very long hyphae (asterisk) and individual rounded yeast-like structures (arrow). **c** Fungal shapes are markedly pleomorphic. Intracellular organisms within macrophages appear ghost-like with pallor and minimal internal structure (asterisk). There are many neutrophils and rare plasma cells. **d** Numerous intracellular non-staining ghost-like organism outlines are noted in a macrophage (asterisk), while irregular segment shape and size appear to be a hallmark of this organism. Scale bar = 10 μm

Cytology slides were stained on an automated stainer (Wescor Aerospray Hematology Stat Model 7122, Wescor, Inc., Logan, UT, USA) with a Wright’s giemsa stain. Cytologic evaluation revealed a highly mixed inflammatory cell population composed mostly of non-vacuolated macrophages with frequent well-preserved neutrophils and infrequent plasma cells. Macrophages appeared singly and in clusters, were often quite large (up to 25 μm) and frequently multinucleated. They had abundant lightly basophilic cytoplasm, occasionally with non-staining intracellular fungal organisms. One slide had a purely macrophage population but also contained the fewest organisms, which were predominantly intracellular and indistinct. On all other slides, there were large numbers of extracellular fungal elements present. Most were non-pigmented septate fungi with irregular walls, short irregularly sized septa, and distinct non-staining wall. Round and larger shapes resembling yeast were also present (Fig. 1a-d). A hub-and-spoke pattern was occasionally noticed with longer hyphae arising from larger rounded yeast-like structures. Bacteria were not identified. The cytological diagnosis was fungal granulomatous inflammation.

**Fig. 2:**
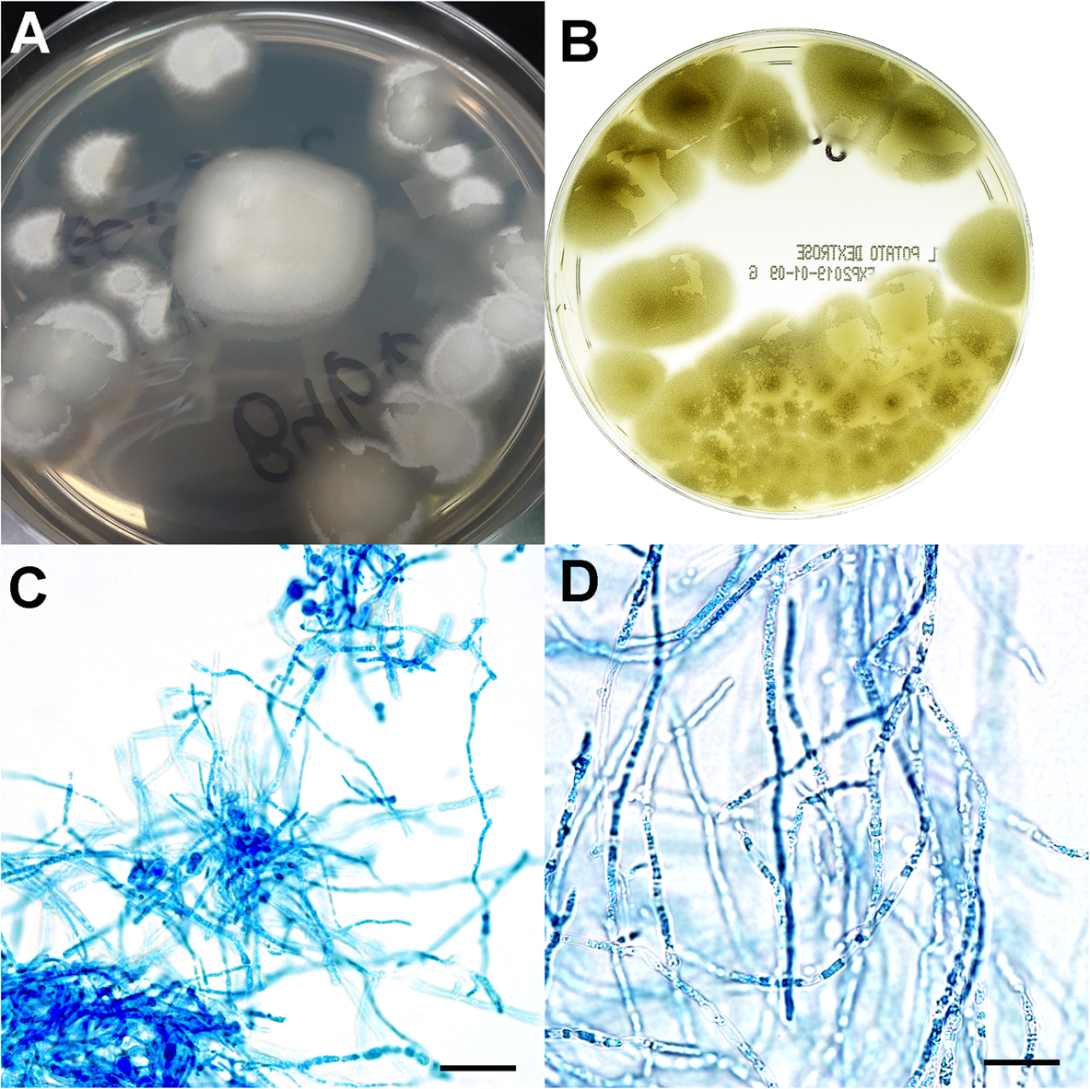
Colony morphology and microscopic features of *Mycoleptodiscus indicus* associated with subcutaneous infection in a cat. **a** *M. indicus* colonies after 2 days of incubation on Sabouraud dextrose agar at 25 °C. At 35 °C, colony morphology was similar. **b** *M. indicus* colonies after 5 days of incubation had brown-yellow pigmentation. **c** Microscopic features of the septate and branched hyphae. Scale bar = 25 μm. **d** Hyphae appeared branched and septate. Scale bar = 10 μm. Slide culture preparation stained with lactophenol aniline blue stain

Based on the recommendation of the cytology report, a repeat aspiration collected from the lesion was submitted 3 days later for fungal culture to the to the Athens Veterinary Diagnostic Laboratory (University of Georgia, USA). After 2 days of incubation at 25 °C and 35 °C, a fast-growing mold was isolated on Sabouraud dextrose agar, with and without chloramphenicol (BD BBL, Franklin Lakes, NJ, USA), and on potato flake agar (Remel, San Diego, CA, USA). The morphology and colony growth rates were similar on all agars and under both temperature conditions; both appeared circular with a white fleecy surface and a light beige reverse (Fig. 2a). After 5 days of incubation, colonies with a brown-yellow pigment appeared on agar (Fig. 2b). Microscopically, hyphae appeared septate and branched on a slide culture preparation mounted in lactophenol aniline blue stain (Fig. 2c-d). Despite daily observation, conidia production was not identified throughout the 3 weeks of incubation on Sabouraud and potato agar at 25 °C and at 35 °C.

DNA was obtained from the isolate grown on Sabouraud dextrose agar as well as from the fine-needle aspirate using a commercial kit (ZR fungal/bacterial DNA MiniPrep, Zymo Research, Irvine, CA, USA) according to the manufacturer’s instructions. Conventional pan-fungal PCR was performed targeting the internal transcribed spacer (ITS) region [9] and the D1/D2 region of the large subunit of the *28S ribosomal RNA* gene [10]. *Aspergillus niger* ATCC 16404 was used as a positive control. PCR products from both genes (*ITS *and *28S ribosomal RNA* gene) were purified and submitted to Georgia Genomic Facility (Athens, GA) for Sanger DNA sequencing as previously described [11, 12]. BLAST analysis (http://www.ncbi.nlm.nih.gov/BLAST) and the CBS-KNAW fungal database (http://www.westerdijkinstitute.nl/collections/) were used on each sequence to identify related fungal sequences. Pan-fungal PCR assays were positive from both the fine-needle aspirate and the agar-grown isolate. The amplified sequences were 99% homologous to sequences from *M. indicus* available in online databases. Due to the rarity of *M. indicus* reports in the literature, we repeated the fungal culture, PCR assays and sequencing from the same fine-needle aspirate. The second analysis on all diagnostic modalities yielded the same results and confirmed the presence of *M. indicus*.

The cat was started on half a 100 mg oral tablet (50 mg) twice-daily fluconazole treatment for 60 days, and the lesion fully resolved without recurrence or sequela. Surgical resection was not performed. The disease-free interval from the time of cessation of antifungal therapy to last follow up was approximately 10 months.

**Fig. 3:**
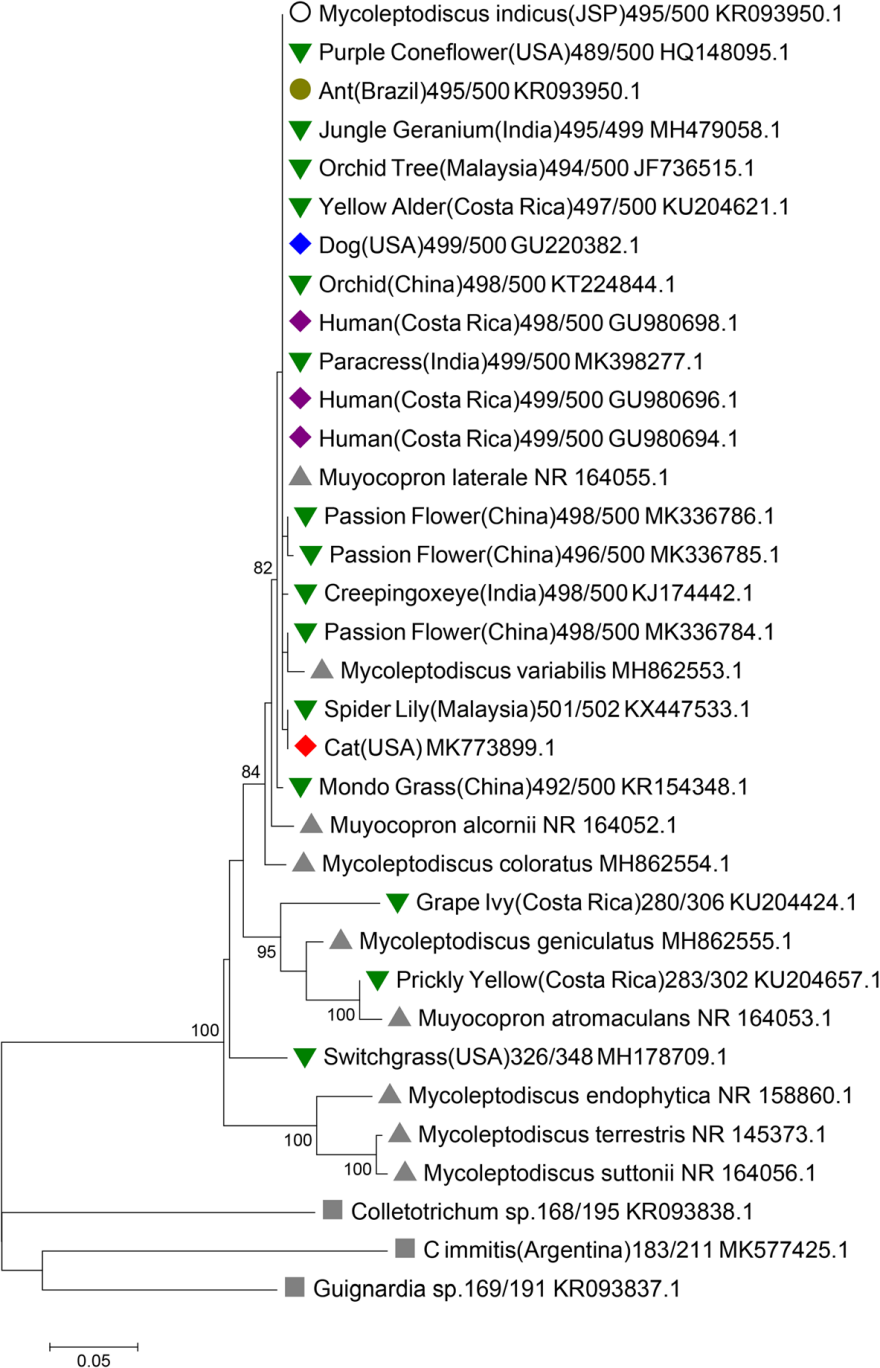
Phylogenetic relationships among *Mycoleptodiscus indicus* strains based on nucleotide sequences of the internal transcribed spacer (ITS) region. Phylogenetic tree contains ITS nucleotide sequence from the feline investigated in this study (accession number: MK773899.1) and 33 ITS gene region sequences downloaded from GenBank. *Coccidioides immitis*, *Guignardia* spp. and *Colletotrichum* spp. were included as the outgroup. The phylogenetic trees were inferred using the Maximum Likelihood method based on the Tamura-Nei substitution model predicted by Jmodeltest and constructed by PhyML. The percentage of trees in which the associated taxa clustered together in the bootstrap test (1000 replicates) is shown next to the branches

Phylogenetic analysis was performed to determine the possible epidemiological and ancestral relation to other known clinical cases of *M. indicus.* Phylogenetic trees were built using the ITS sequence from this study (Genbank accession number: MK773899.1) and 33ITS nucleotide sequences retrieved from GenBank. The sequence alignment was performed using a Multiple Alignment using Fast Fourier Transform (MAFFT) software [13], and poorly aligned positions were trimmed for phylogenetic analysis. The phylogenetic trees were inferred using the Maximum Likelihood method based on the Tamura-Nei substitution model predicted by Jmodeltest [14] and constructed by PhyML [15] with 1000 bootstrap interactions. The initial tree for the heuristic search was obtained automatically by employing Neighbor-Join and BioNJ algorithms to a matrix of pairwise distances which were then estimated using the Maximum Composite Likelihood approach. Phylogenetic analysis identified the sequence clearly aligned in close proximity to the strain that infected a dog in the United States and a human infection case (Fig. 3). Sequences from human and domesticated animals also aligned in close proximity with *M. indicus* sequences from plants (Fig. 3). In addition to the overall high degree of conservation between all sequences, the ITS sequence from the cat had fewer polymorphisms when compared with the ITS sequence polymorphisms found in an ornamental plant species, *Crinum asiaticum* (one single polymorphism) (Additional file 1). This illustrates the high similarity between the cat and the plant ITS sequences. Human and dog sequences had 3 polymorphisms (Additional file 1). Genbank accession numbers: *Crinum asiaticum* KX447533.1; Dog GU220382.1; Cat MK773899.1; Human GU980694.1.

## Discussion and conclusion

*M. indicus* is a rare cause of human and animal infections, though it is a well-recognized plant pathogen [1]. There are few reports in the literature of *M. indicus* infection in humans and only one report in a dog [3, 5–8]. *M. lateralis*, a similar species to *M. indicus,* was reported to cause subcutaneous phaeohyphomycosis in a cat from Australia [16]. This report was published in a conference proceeding, which was difficult to access in the literature. A recent study showed that *M. indicus* and *M. laterale* have insufficient genetic differences to consider them distinct species [2]; however, the National Center for Biotechnology Information (NCBI) taxonomy stills lists them as two different species. There are no other publications available to clarify this issue.

Skin lesions appear to be the most common clinical presentation of this mycosis. The clinical features noted in this cat were very similar to the reported case of *M. indicus* in a dog [8]; both patients had subcutaneous infection in a swollen limb. However, the dog was immunosuppressed and died 2 months after his diagnosis, while the cat in this case report did not appear to be immunosuppressed and continues to do well without ongoing treatment after a single course of antifungal therapy.

Prompt clinical resolution of *M. indicus* infection seems to be associated with the immune-competent status of the patient. An immunocompetent person developed *M. indicus* synovitis and osteomyelitis after being scratched by tropical plants while travelling in Costa Rica. His infection resolved with simple debridement of the affected areas and without any antifungal therapy [7]. In contrast, treatment of *M. indicus* infection in immunocompromised human patients appear to be challenging and require a combination of surgical debridement and antifungal therapy for resolution. Three cases of *M. indicus* skin and joint infections in immunocompromised humans were resolved by debridement with or without months of systemic antifungal therapy [5, 6, 8]. The dog and the cat cases are consistent with the human reports, in that the immunocompetent cat infection appeared to resolve completely and quickly, while the immunocompromised dog patient did not.

The laboratory identification of *M. indicus* is challenging. This is due to the rarity of this fungus, lack of cytologic morphological description, its fastidious nature, and the need for specialized culture conditions to induce conidia production. In our case, the microscopic identification of *M. indicus* colonies and hyphae stained with lactophenol aniline blue stain caused initial confusion due to its similarity to *Coccidioides immitis.* The white or buff color and morphology of colonies resembled *C. immitis*, as did the hyphae in the absence of arthroconidia [17]. However, the direct cytological analysis from the fine needle aspirate revealed markedly pleomorphic organisms that varied from yeast-like to septate and branched hyphae. This morphology was unique and did not resemble any known fungal pathogens readily identified on cytology, including *C. immitis*. DNA sequencing contributed to a rapid diagnosis, overcoming the challenging morphological identification. The rapid diagnosis facilitated the early intervention and may have contributed to the successful clinical outcome. Specific treatment guidelines do not exist for *M. indicus* infection, therefore the selection and dosage of antifungal treatment using azoles was based on first principles and the partial resolution of lesion in the canine case report [8].

*Mycoleptodiscus* spp., have been isolated from soil and endophytes from a variety of plants worldwide and potentially serve as its main natural reservoir [1, 2, 4]. The origin of the *M. indicus* strain that infected the cat in this study remains unclear. Infection of a distal limb would be consistent with accidental implantation from a plant or secondary to a scratch or wound, as could occur with animals walking on lawns or other natural surfaces. The strain infecting this cat was closely related to the *M. indicus* strains that infected a dog and humans in North America [6–8]. Sequences from humans, dog and our patient are similar to fungal isolates obtained from infected plants. This supports the hypothesis that this fungus, which is well adapted to plants, may have initially evolved from plants to adapt and infect different mammals. It also supports previous reports suggesting that plants were the main source of *M. indicus* infection in humans [3, 6, 7]. *M. indicus* in the present report could also be considered an accidental opportunistic pathogen after breaching the skin barrier and being introduced in the subcutaneous tissues of the cat. Future studies could investigate phylogenetic differences between pathogenic and non-pathogenic isolates from mammals and plants.

A recent study presented a molecular phylogeny and revision of the *Mycoleptodiscus* genus using a wide collection of isolates [2]. Interestingly, *M. indicus* was placed in a lineage with species of *Muyocopron laterale*, therefore, there is a possibility that *M. indicus* name may be changed to *Muycopron laterale*.

In summary, to our knowledge, this is the first report of subcutaneous *M. indicus* infection in a cat. Unlike other reports of this infection in people and the single case reported in a dog, the cat was not immunocompromised, and antifungal therapy with fluconazole resolved the infection. The origin of the strain infecting the indoor-outdoor cat remains unclear, but phylogenetic analysis supports the hypothesis that plants are potential sources of *M. indicus* infection to humans and animals. As only one gene was used in this analysis, additional evaluation with more samples and gene markers would be needed to better evaluate this hypothesis. Veterinary clinicians and diagnosticians should be aware of the potential for immunocompetent animals to become infected with *M. indicus,* its unusual but characteristic cytological appearance, and the need to combine pathology, microbiology and molecular laboratory testing to optimally diagnose and treat opportunistic fungal infections.

## Supplementary information

**Additional file 1 **Comparative alignment between *Mycoleptodiscus indicus* ITS sequences from the cat, dog, human and *Crinum asiaticum* (Spider Lily, plant). The ITS sequences alignment highlights the similarity between the cat and the plant sequence. Black shaded regions represent conserved regions and white shaded are the variable sites. Genbank accession numbers: Spider Lily KX447533.1; Dog GU220382.1; Cat MK773899.1; Human GU980694.1.

## Data Availability

All data generated or analyzed during this study have been included in this published article. The *M. indicus* sequence investigated in this study is available on Genbank under the following accession number: MK773899 (Link: https://www.ncbi.nlm.nih.gov/nuccore/MK773899).

## Abbreviations

ATCC: American Type Culture Collection
BLAST: Basic Local Alignment Search Tool
ITS: Internal Transcribed Spacer
MAFFT: Multiple Alignment using Fast Fourier Transform
PCR: Polymerase Chain Reaction

## References

[1] Sutton BC (1973). *Pucciniopsis*, *Mycoleptodiscus* and *Amerodiscosiella*. Trans Br Mycol Soc.

[2] Hernández-Restrepo M., Bezerra J.D.P., Tan Y.P., Wiederhold N., Crous P.W., Guarro J., Gené J. (2019). Re-evaluation of Mycoleptodiscus species and morphologically similar fungi. Persoonia - Molecular Phylogeny and Evolution of Fungi.

[3] Padhye AA, Davis MS, Reddick A, Bell MF, Gearhart ED, Von Moll L (1995). *Mycoleptodiscus indicus*: a new etiologic agent of phaeohyphomycosis. J Clin Microbiol.

[4] Spear ER (2017). Phylogenetic relationships and spatial distributions of putative fungal pathogens of seedlings across a rainfall gradient in Panama. Fungal Ecol.

[5] Garrison A, Procop G, Vincek V, Moon J, Morris M, Doblecki-Lewis S, Cleary T, Brust D, Rosa-Cunha I (2008). A case of subcutaneous *Mycoleptodiscus indicus* infection in a liver transplant recipient successfully treated with antifungal therapy. Transpl Infect Dis.

[6] Koo S, Sutton DA, Yeh WW, Thompson EH, Sigler L, Shearer JF, Hofstra DE, Wickes BL, Marty FM (2012). Invasive Mycoleptodiscus fungal cellulitis and myositis. Med Mycol.

[7] Dewar CL, Sigler L (2010). Fungal arthritis of the knee caused by *Mycoleptodiscus indicus*. Clin Rheumatol.

[8] Metry CA, Hoien-Dalen PS, Maddox CW, Thompson EH, Sutton DA, Romanelli AM, Wickes BL, MacNeill AL (2010). Subcutaneous *Mycoleptodiscus indicus* infection in an immunosuppressed dog. J Clin Microbiol.

[9] Ferrer C, Colom F, Frasés S, Mulet E, Abad JL, Alió JL (2001). Detection and identification of fungal pathogens by PCR and by ITS2 and 5.8 S ribosomal DNA typing in ocular infections. J Clin Microbiol.

[10] Kwiatkowski NP, Babiker WM, Merz WG, Carroll KC, Zhang SX (2012). Evaluation of nucleic acid sequencing of the D1/D2 region of the large subunit of the 28S rDNA and the internal transcribed spacer region using SmartGene IDNS software for identification of filamentous fungi in a clinical laboratory. J Mol Diagn.

[11] Stimmelmayr R, Rotstein DS, Maboni G, Person BT, Sanchez S (2018). Morbillivirus-associated lipid pneumonia in Arctic foxes. J Vet Diagn Investig.

[12] Kokosinska A, Maboni G, Kelly KM, Molesan A, Sanchez S, Saliki JT, Rissi DR (2019). Lymphoplasmacytic meningoencephalitis and neuronal necrosis associated with parvoviral infection in cats. Vet Pathol.

[13] Katoh K, Standley DM (2013). MAFFT multiple sequence alignment software version 7: improvements in performance and usability. Mol Biol Evol.

[14] Darriba D, Taboada GL, Doallo R, Posada D (2012). jModelTest 2: more models, new heuristics and parallel computing. Nat Methods.

[15] Guindon S, Dufayard J-F, Lefort V, Anisimova M, Hordijk W, Gascuel O (2010). New algorithms and methods to estimate maximum-likelihood phylogenies: assessing the performance of PhyML 3.0. Syst Biol.

[16] Hull W, McNamara T, Ireland G (1997). Subcutaneous phaeohyphomycosis due to *Mycoleptodiscus lateralis* in a cat. XIII Congresso Estadual de Medicina Veterinária, II Congresso de Medicina Veterinária do Cone Sul: 1997.

[17] Ampel NM. The diagnosis of coccidioidomycosis. F1000 Med Rep. 2010; 2:2. 10.3410/M2-2 PMC2948390 20948866

