# Supplementary figures and images for: Laboratory diagnostics, phylogenetic analysis and clinical outcome of a subcutaneous Mycoleptodiscus indicus infection in an immunocompetent cat
Source: BMC Vet Res. 2019 Oct 21;15:354. doi: 10.1186/s12917-019-2132-1 (PMC6805521; doi:10.1186/s12917-019-2132-1)

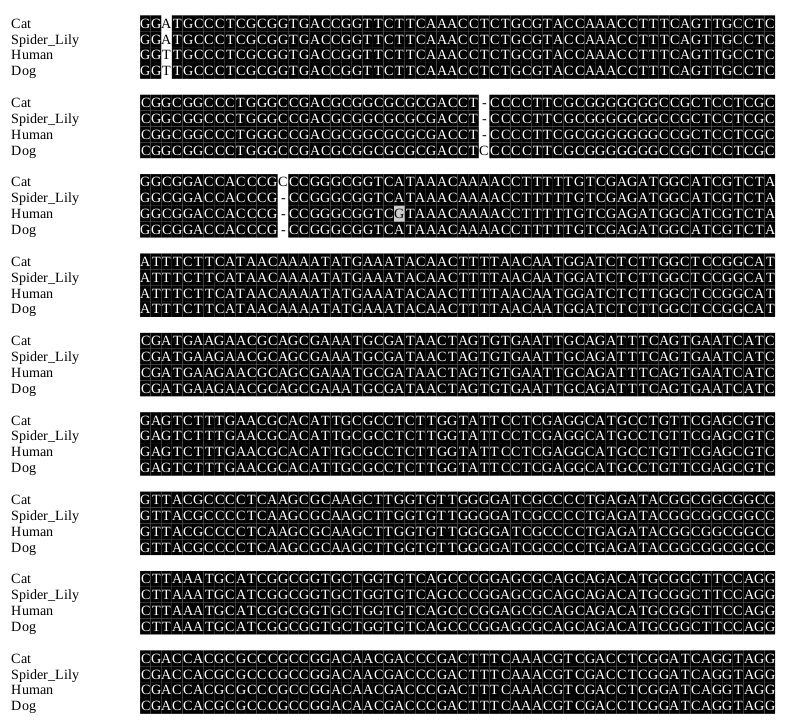

Supplement: Supplementary file 1 — Additional file 1 Comparative alignment between Mycoleptodiscus indicus ITS sequences from the cat, dog, human and Crinum asiaticum (Spider Lily, plant). The ITS sequences alignment highlights the similarity between the cat and the plant sequence. Black shaded regions represent conserved regions and white shaded are the variable sites. Genbank accession numbers: Spider Lily KX447533.1; Dog GU220382.1; Cat MK773899.1; Human GU980694.1. [file 12917_2019_2132_MOESM1_ESM.tif]
